# When We Study the Ability to Attend, What Exactly Are We Trying to Understand?

**DOI:** 10.3390/jimaging8080212

**Published:** 2022-07-31

**Authors:** John K. Tsotsos

**Affiliations:** Department of Electrical Engineering and Computer Science, York University, Toronto, ON M3J 1P3, Canada; tsotsos@yorku.ca

**Keywords:** visual attention, human vision, computational vision, visual function, functional vision, selective tuning, executive control

## Abstract

When we study the human ability to attend, what exactly do we seek to understand? It is not clear what the answer might be to this question. There is still so much to know, while acknowledging the tremendous progress of past decades of research. It is as if each new study adds a tile to the mosaic that, when viewed from a distance, we hope will reveal the big picture of attention. However, there is no map as to how each tile might be placed nor any guide as to what the overall picture might be. It is like digging up bits of mosaic tile at an ancient archeological site with no key as to where to look and then not only having to decide which picture it belongs to but also where exactly in that puzzle it should be placed. I argue that, although the unearthing of puzzle pieces is very important, so is their placement, but this seems much less emphasized. We have mostly unearthed a treasure trove of puzzle pieces but they are all waiting for cleaning and reassembly. It is an activity that is scientifically far riskier, but with great risk comes a greater reward. Here, I will look into two areas of broad agreement, specifically regarding visual attention, and dig deeper into their more nuanced meanings, in the hope of sketching a starting point for the guide to the attention mosaic. The goal is to situate visual attention as a purely computational problem and not as a data explanation task; it may become easier to place the puzzle pieces once you understand why they exist in the first place.

## 1. Introduction

Imagine that one morning—perhaps it is a dream—you find yourself at the top of a high mountain on a sunny cloudless day overlooking a vast flat plain that stretches as far as the eye can see. On this plain, all the papers or books ever written focused on the topic of attention are arrayed, whether in humans, animals, or machines. You are surprised to discover that you also have the abilities needed to read all of these papers and to assimilate their contents before the sun sets. This may sound ridiculous, but consider this. If papers are printed on standard letter size paper, collated and stapled, each needs about 600 sq.cm. of area, with the thickness depending on the number of pages. A human standing on a flat plain can see about 5 km to the horizon. From a mountaintop, one sees more. The area of a circular plain with a radius of 5 km is about 8 × 10^11^ sq.cm. One could thus lay out over 1 trillion papers, likely enough. As for your ability to read and assimilate them in a dozen hours, a bit more imagination might be needed. As you watch the crimson setting of the sun, and with this new perspective, what do you conclude? I will focus on visual attention, perhaps one of the best studied aspects of attention, with the visual cortex being perhaps the most studied part of the biological brain and computer vision being one of the most active areas of machine intelligence research and application.

Your view of the literature should have shown you that, by a large majority, visual attention is studied separately from its natural embodiments. I contend that this distorts its true nature. One way to look at this is to consider the characterization of *visual function* and *functional vision*:

“The complete assessment of an individual’s vision-related abilities requires the consideration and characterization of both visual function and functional vision. … *visual function* describes how well the eyes and basic visual system can detect a target stimulus. By varying a single parameter at a time (for example, the size of the target), testing is typically carried out in a repeated fashion under controlled testing conditions until a threshold of performance is obtained. … *functional vision* refers to how well an individual performs while interacting with the visual environment. That is to say, how their vision is used in everyday activities. Characterizing functional vision involves the assessment of multiple and varying parameters captured under complex, real-life conditions.” [1].

These seem to be sensible ways to add emphasis to embodiment, but perhaps not as precise as they might be. I would add this clarification. The assessment of visual function is performed by the assessor by taking control of what is seen by the subject and restricting the range of their possible responses to what is seen. By contrast, in functional vision, the subject determines what is seen and can respond in any appropriate way with existing or novel behaviors. This is not intended as a strict dichotomy; the point is to highlight the full breadth of visual intelligence that an experimenter might study. There are many sub-categories of tasks in each class. Functional vision in this light is not simply a strategic deployment of visual function tasks. Human (or animal) visual abilities did not evolve by looking at two-dimensional, cropped scenes on a flat surface while sitting in a chair, and the way we view vision research should reflect this.

One mostly sees attentional studies as focusing on visual function in both the computational and biological disciplines, while studies of functional vision seem far fewer and less conclusive. Visual function studies typically take on the order of many milliseconds to a few seconds and subjects perform many such trials. Functional vision tasks can have arbitrary timelines and nature; however, temporal duration is not the main demarcation between these (see Sanocki and Lee [2], who look into different timelines). Experimenters sometimes use free-viewing as a task and this may sound like functional vision as described here. It is not. Fully free observation in animals is likely impossible whereas in machine vision it is easy. You can imagine free viewing perfectly well in a machine where one can design the machine to be entirely sense driven. In animals, unless one can eliminate any use of what one has in memory, free-viewing is never free from prior influences, priorities and knowledge.

What is very clear is that humans have the ability to actively find the information they need to solve a task; they are not passive receivers of information. When we search for something, we do not wait motionless, eyes fixated on one spatial location, for our target to perhaps walk into our line of sight (i.e., as would a surveillance camera system, or the vision system of a barnacle). However, our experimental literature mostly takes exactly this form, and perhaps this is necessary to help with reproducibility and to reduce ambiguities in how results may be interpreted. Humans and the vast majority of animals are active observers. We acquire data by sensing parts of our world as it is required, while always being vigilant to our environment, and we reason about its role in fulfilling tasks.

Whereas technical as well as colloquial uses of the word "attention" make it sound like everyone knows what it means, I will argue that I really do not think everyone knows what attention is nor is it clear what the goals are when studying attention. From your mountaintop perch, you too should agree. However, I do understand that everyone’s first impulse will be to think that we know a great deal about attention; there are so many experiments, so many models, so many good theories, and so on (for example, [2,3,4]), and you would not be mistaken. My goal is orthogonal to all this. Consider what your thoughts might be while scanning the vast literature. You notice that what is studied and how it is approached varies widely. You will no doubt notice a great variety of connections among works, many times unstated by the authors but they pop out to you because you are taking a high altitude snapshot of the field. You also see many inconsistencies and disagreements and contradictions. It is often also difficult to know what exactly has been sufficiently replicated to believe it is fact or sufficiently refuted to believe it is false. How can this be with so many experiments by so many smart and dedicated scientists? I am convinced these issues arise because all are not studying the same form of attention. In other words, even if restricting the focus to visual attention, there is no agreement on what the problem of attention might be, let alone how to approach it. Attention is not a single phenomenon; it is a complex of phenomena not all relevant for every single kind of stimulus, deployed dynamically as the need dictates at the time. The use of the qualifier ’dynamically’ here is intended to emphasize that for any particular stimulus input, choices must be made as to which attentional processes are needed, how each needs to be initialized, in what sequence they are executed, and how their outcomes are handled. Perceptual selection processes that may be always active, although important, are not in this group.

I will discuss this in two parts, each dealing with themes that your bird’s-eye view of the literature should have made evident. Let me first clarify the manner of presentation. I will try to convey my points focused at a single level of abstraction. The levels of description relevant for describing the brain, its function, its anatomy, its computational aspects, have appeared previously, perhaps best shown in Marr [5], Churchland and Sejnowski [6], Wilson [7], and Ballard [8]. I will follow Marr, who described “the three levels at which any machine carrying out an information-processing task must be understood”:

- *Computational theory*: What is the goal of the computation, why is it appropriate, and what is the logic of the strategy by which it can be carried out?
- *Representation and algorithm*: How can this computational theory be implemented? In particular, what is the representation for the input and output, and what is the algorithm for the transformation?
- *Hardware implementation*: How can the representation and algorithm be realized physically?

Each of the cited authors stresses the problem of how to choose at which level of abstraction to perform an experiment or analysis. They also argue that a complete understanding can only come from the combined understanding of all levels. I agree, but will add that currently, we need to first agree on what problem we are trying to understand. Thus, I will stay at the computational theory level exactly because I feel it is important that we, as a community, agree on the problem that we wish to solve. Furthermore, if one attempts to describe this problem and bounces between these levels while doing so, the statement becomes confused and easy to argue against because the descriptions at the different levels will be incomplete. Justifying some aspect of the computational theory by saying that it is true because there is some anatomical or behavioral evidence for it opens the door to it later being refuted when we discover that evidence is untrue or more nuanced. The computational level description must be valid for any implementation, it must be logically consistent on its own, and it must stand on its own.

To this point, something has been missing. I thus add a small qualification to Marr’s levels, namely my own *complexity level* [9,10]. The reason I do this is because Marr, although a superb neuroscientist, was not trained in computer science. Computer scientists realize that there are always resource constraints. However, this is not new in the attention context. Kahneman pointed this out, but not quantitatively nor formally, in his general purpose limited resource model [11]. Marr’s computational level included “what is the logic of the strategy by which it can be carried out”? Describing a logic that can never be realized is not a useful exercise. Over the years, there have been a huge number of computational problems described that are formally not tractable or even decidable, many that may surprise yet find their way into computational level proposals. For more detail on these concepts, see [10,12]. As a result, my complexity level requires that proposed strategies are realizable in actual brains or machines; it is a level of analysis that is orthogonal to Marr’s. In this paper, it is primarily applied at Marr’s computational level, the level where the nature of the problem and its solution are described. In this way, it is required for any possible realization of a solution. The alternate is to apply it at the implementation level, which is specific for a particular realization (perhaps realizable in machines but not in animals, as much of modern machine learning seems to be). It is more powerful in the former case.

I will convey my points as a journey from first principles to conclusion; I will not give you a glimpse of the end of the journey in advance. Reasoning from first principles is a problem-solving technique that requires you to break down a complex problem into its most basic, foundational elements. The idea is to ground yourself in the foundational truths and build up from there. Think of it as descending the mountain and walking from its foothills, across the valley to the horizon and only upon reaching the horizon do you have a full understanding.

## 2. Attention Is Needed Because There Is Too Much Sensory Input

Perhaps the most common and persistent theme in this entire literature is that humans, as well as machines, need attention because the amount of sensory input to process is too large. The direct instantiation of this for vision is that the output from the large number of photoreceptors in the retina is too much for the brain to process. Thus, one solution is for the brain to process only portions at any point in time. In machines, it would be that the size of an image in terms of pixels is too large. Typical images for computer vision have spanned all sizes from 28 × 28 (the MNIST data set) up to gigapixel images. The first major successes of the modern era of computer vision (the AlexNet object categorization system) were demonstrated on 256 × 256 pixel images (65,536 pixels in total; but then resized to be smaller, 224 × 224 pixels ). These sizes are really not large and the evolution of computing power (as evidenced by tracking its explosive growth, [13]) now provides plenty of power for these numbers of pixels and larger. In fact, many attribute the current success of machine learning methods to the concurrent availability of sufficient computer power. The human retina has about 126,000,000 photoreceptors so those machine images are about 2000 times smaller. For humans, evolution has taken care of things; the human brain can handle the number of its input photoreceptors with no apparent difficulty. It is important to highlight that when the first statements regarding too much sensory input to process were being made (in the 1950s), they were totally true with respect to computer power; it was also difficult to quantify brain power at the time (and it still is) so the statement was easily and understandably accepted. The problem is that the assertion has outlived its usefulness.

**Figure 1:**
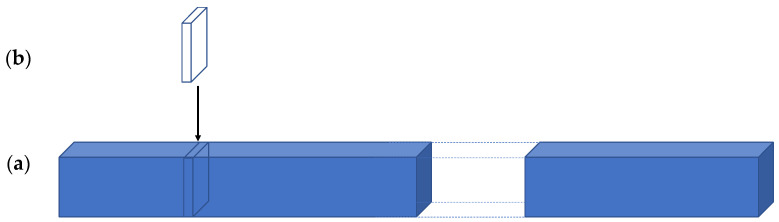
A depiction of the space of all possible discernably different images. (**a**) Suppose that part **a** is a depiction of a stack of upright images and assume that, for the scale in the figure, the stack represents a billion images. (**b**) The stack in **a** is taken from the space of all possible different images represented by the extended rectangular cuboid whose length is unknown.

More important than the number of pixels are two other measures. The first is most relevant for modern machine learning approaches to vision. Learning from data depends on one foundational assumption: the answer one seeks is in the data and only in the data. Let us say that the goal is to learn attentive processing from observed human data. Consider the following (Figure 1).

Figure 1a is a depiction of a stack of upright images, and assume that, for the scale in the figure, the thickness of the stack represents a billion images. (As of 11 March 2021, the ImageNet database contained 14,197,122 images of many sizes, averaging 469 × 387 pixels; https://www.image-net.org/update-mar-11-2021.php accessed on 30 July 2022). This stack is taken from the space of all possible different images represented by the extended cuboid, Figure 1b, whose length is unknown. What is the number of all possible discernably different images? We first would need to set image size; do we consider retina-sized images or the usual computer vision sizes? Pavlidis [14] goes through an analysis and using 1200 × 1200 pixel images, has estimated the lower bound on this image number to be anywhere from 10^25^ to 10^400^. If one considers two retina size images (each with over 80 times Pavlidis’ number of pixels), the higher of these bounds might be much too small. Using the smaller estimate at the scale in the figure, if the stack in Figure 1a is 1 cm wide, the extended stack of Figure 1b becomes 10^11^ kilometers long! For comparison, this is about 658 times the distance of the earth to the sun. It is not difficult to argue against the specifics of such counts; but even if one takes any objections into account, there is no doubt that the space of all potential images that may be viewed is unimaginably large. This might provide an estimate appropriate for tasks on single images, but if we add real-world vision, we need to consider sequences of images of arbitrary (yet finite) length and thus, these numbers are actually far too small. One can also count images differently and ask how many images might any one person actually see in a lifetime? We know that it takes about 250 ms to prepare and execute a visual saccade. Each saccade leads to an image to be processed. How many saccades could we have in a normal lifetime? A period of 90 years contains 2,838,240,000 seconds. Our eyes are closed about one-third of that time, but we can make four saccades per second during the time they are open, or see 7,568,640,000 images. The world of course is both continuous and in motion, not discretized as this may be suggesting. Say that 100 images per second is sufficient (knowing that normal human-created movies are displayed at 24 fps). During our eyes-open lifetime, this means we process about 1.9 × 10^11^ images. This very large number is still many orders of magnitude too small to sensibly sample the space of all discernably different images. The distribution of images we can actually see is far from uniform across all the possible dimensions of visual experience because in those 90 years, we could not possibly see every variation of every visual parameter. What the dimensions of visual experience might be are unknown.

The numbers are not the point here. The point is that they are too large to ever imagine that any sampling of this space could be statistically sound. Current systems sample from the known, and in machine vision, seem to care little about ensuring populations are unbiased. Visual attention, or attention more generally, is not fully known with respect to either its observable characteristics nor its internal processing methods, so thinking about how one might sample the space is daunting. Generalization methods might extend their reach a bit or fill in small gaps but certainly cannot create samples that do not fit the original population distribution. This means that the best models based on limited data sets are only explanatory and not predictive (see [15]). In order for a model to be predictive, it must be forward-looking, predicting new and counter-intuitive observations that do not flow from the currently available data, i.e., that are not consistent with its training population. Thinking that a data-learning strategy for the general problems of both visual function and functional vision is sufficient seems inappropriate.

The second point to make in this section is that, even if you think these numbers are unreasonable for whatever reason, the grouping combinatorics within each image have an exponential nature so the conclusion remains (if an image contains *P* pixels, the number of possible ways of groupings pixels into objects is *2^P^*). If one makes each location represent more than a binary quantity (as would be normally true with color, depth, time-varying properties represented at every spatial location), the number of groups becomes *2^PM^*, where *M* is the number of features (assuming subsets of features are relevant for defining a group). This is even worse. Just for fun, if *P* = the number of photoreceptors and *M* is, say, five, the number of groupings is 2^5*126000000^, a bit too much to deal with in any manner. The image combinatorics lead to the intractability that triggers the need for attention. Earlier, the term *first principles* was mentioned as a guide for this presentation; the content of this section demonstrates exactly what was meant.

It is often difficult to imagine what an intractable problem instance might look like. Imagine you decided to stay on your mountaintop after sunset to view the stars in the dark clear sky. The full array of stars above you stretch from horizon to horizon and you remember that, as a child, you would look at the sky and wonder about the mythical constellations. Suppose you were asked to find a group of stars that formed a hexagon with equal length sides. You begin your search, and soon realize you are checking every grouping of stars you can construct for its overall configuration and side lengths. How many groupings are there of *N* stars? The answer is *2^N^*, the powerset of *N.* How many groupings of six stars out of *N* stars? The answer is *N*!/(6!(*N* − 6)!). *N* does not need to be an unimaginably large number, but the number of stars visible in the night sky is pretty large. Even if it were only about a thousand, the number of potential subsets, *2^1000^* = 1.07 × 10^301^, and the number of groupings of six stars is 1000!/(6!(1000 – 6)!) or about 1.4 × 10^15^ which seems too large to handle. You do not know what size the hexagon will be, what orientation it might have, where it can be found, and what you might use to restrict the group of stars that you consider. This is what an intractable instance looks like. This is similar to the argument made by Stockmeyer and Chandra [16], where they show that for some computational problems that are well-defined and decidable, even a universe full of proton-sized computers operating at the speed of light would take longer than the age of the universe to complete a solution. They are thus beyond computation—intractable—in their general case. The intractable problem needs too many physical resources for computation and too much time to complete the computation.

The apparent ease or difficulty of any task, to a casual observer, often is not directly connected to its formal tractability. The number of computational problems that are mathematically proven to be intractable is quite large [12] and of those, the number that are directly related to intelligent behavior or cognitive science is significant [17]. Most are tasks we all perform many times a day so it is easy to ignore the formal proofs.

So if we perform them daily, does this not contradict the intractability claim? Theory tells us that such provably intractable problems may never be solved using a single, optimal solution that applies to all possible instances. Theory tells us that it is more likely that we need to solve subsets of instances separately, to consider approximation methods, and allow for optimal solutions for special cases and suboptimal ones for others. In other words, theory tells us to partition the problem space and select sets of instances to solve in different ways with potentially differing levels of performance (accuracy as well as time to complete). This is what we do every day: achieve the best result we can in the time provided. Now, we are entering the realm of what Marr termed “the logic of the strategy”, which can be applied to a problem. 

One possible problem instance set is those tasks for which hints can reduce the required resources. That an advance cue can improve accuracy has been known ever since Posner’s classic work [18], at least. In a formal manner, our past work has shown how a little task knowledge can make exponentially important reductions to the search space, both theoretically and empirically [19,20]. Consider our constellation example again. Suppose that you know that the hexagon in the sky that you seek has a width of 20° of visual arc. How does this change the calculation? We need to compute the value of N!/(6!(N − 6)!) where N is still 1000 but where the acceptable groupings of six have the characteristic that no distance between any two stars is greater than 20°. For the sake of the back-of-envelope calculation, assume that the stars are uniformly spread over the sky. The sky can be approximated by a half sphere whose surface area is about 1.57 × 10^8^ sq.m. (*r* = 5 km, the distance to the horizon that you can see from your mountaintop vantage). Thus, there would be about one star per 1.57 × 10^5^ sq.m. of sky. The 20° constraint tells us that we only need to look at the region centered by each star and check any configurations that include that center star within a 20° radius. A circular patch in the sky (known as a spherical cap of the half sphere) of a 20° visual arc radius has an approximate area of about 9.5 × 10^6^ sq.m., thus containing about 60 stars. There are 1000 stars, each the center of such a 20° radius region, each with about 60 stars. How many groupings of six stars can there be out of 60? The answer is about 5 × 10^7^. Thus, to check each possibility, one checks many groups for each of 1000 stars or about 5 × 10^10^ candidate groups of six stars. This is an improvement of five orders of magnitude compared to the earlier calculation where there was no specific task information. Actually, this over-counts; at the horizon, full discs would overflow below the horizon, so the actual count is smaller still and the apparent benefit larger. To take advantage of task guidance requires that the overall process be adaptable and flexible to such knowledge, i.e., be an active one whose parameters are set dynamically, and the advantages in search space reduction are very clear.

If a cue or task specification makes such a large impact, how does it enter the logic of a solution? Clearly, it must move from outside the system to parts inside the system that it might impact. If the cue is presented verbally or sensorially, it must first be processed and realized as a cue; this likely means that for it to affect any processing it must be applied from more central levels of brain processing to the sensory level. This sounds like what is commonly known as a top-down process. Given the huge improvement in the search that is possible, it is reasonable to conclude that top-down influences are always present, whether they arise from explicit cues and tasks or internal memories.

To sum up, it is true that attention is needed because there is too much sensory input. However, it is not the raw amount of input but the combinatorial nature of grouping image elements that poses the insurmountable challenge that necessitates attention. It is even more difficult when considering functional vision since there can be many different behaviors or interactions with the same visual world. A learning methodology seems to need humanly infeasible amounts of training data, and thus is not an option if we wish to understand human vision. So, the major task is how to partition the total set of possible instances so that each partition is easier to solve separately than the whole. If we wish to understand human visual intelligence, an important constraint is to discover the same partitions that humans use. In machine vision, any partition that satisfies the resource specifics and task will suffice, but it may not behave as humans in all cases. Top-down or task influences help reduce the size of the search space. If there are multiple solution strategies, there needs to be a method to know which one to apply when presented with a particular instance and, of course, to recognize what instance class is relevant at the time. The formal grounding of the discussion in this section takes these points beyond mere opinion; they form part of the foundation of the first principles of the attention problem.

## 3. Attention Selects the Relevant

The other most common theme is that attention is based on the action of selection. Selection relieves any problems posed by either the amount of sensory data or its grouping combinatorics by presenting any computing machinery only with manageable, bite-sized pieces of the data. However, this cannot be the full story; how would anyone know in advance that there was nothing urgent that is not part of a bite? Humans are perfectly capable of spotting events in both near and far periphery that might require urgent response, so there needs to be a parallel route too.

**Figure 2:**
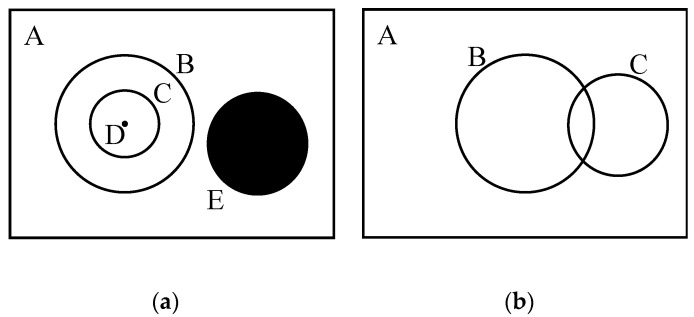
An illustration of the possible types of selection. The Venn diagrams show subsets of some overall population of data A. (**a**) D represents selection of one-of-many. B and C are selections of some-of-many, both are subsets of A but also C is a subset of B, and D is a subset of both. A none-of-many selection would mean an explicit ignoring, illustrated by the set E. (**b**) The selection process can also lead, via separate operations of selecting some-of-many, to the partially overlapping sets B and C. The intersection is a third set which would reflect properties of both B and C.

Consider selection more generally. The most direct interpretation of selection is that something is chosen from among a number of alternatives. This might indeed mean to choose *one-of-many*, but it could also mean to choose *some-of-many* (i.e., to create a subset) or explicitly choose *none-of-many* choices (i.e., to ignore or suppress some or all alternatives), or more. Figure 2 illustrates this.

The vast proportion of the literature tacitly implies that there is only one act of selection required for the solution of any particular visual problem. Given the kind of real-world visual task behavior in the definition of functional vision, there may in fact be many such selections involved to complete any given task. Furthermore, there is no requirement that a selection occurs only in the input space. In other words, the set A of Figure 2 can be the set of image locations but it might also be something else. It might be the set of feature spaces represented in the input (the range of colors or depths). It could represent the entire knowledge base of a system (e.g., every object we know, every place we know, every person we know, etc.). It could be the set of behaviors that one has at their disposal to respond to tasks or situations. Selection can occur within each of these spaces. If the numbers presented earlier with respect to images seemed large, they are far too small if you consider that, for each image, one might ask many questions and receive a different response for each arising from the same input (e.g., [21]). The ability to select the relevant within any of the applicable knowledge sets seems necessary in order to make response a feasible activity. In other words, the number of possibilities of input, task, and response behavior is the product of the three sets.

A selection of some-of-many might be followed by a subsequent selection made on its result. This allows one to form a sequence of attentional operations. If the subsets selected become progressively smaller, perhaps more refined towards a response to a task, then a hierarchical representation could be formed. Such a hierarchy of spatial representations (a layered network) was first proposed by Uhr [22], and was shown to be an important component for dealing with the computational complexity of visual processes in machines and in brain [9,23]. This interpretation opens up the full domain of set theory (the reader who wants a quick introduction to set theory can find it at https://en.wikipedia.org/wiki/Set_theory accessed on 30 July 2022, while the one interested in a more formal and complete description could see [24]). Given a population and two subsets of that population, set theory defines 16 different operations that can specify all partitions shown in the Venn diagram of Figure 2b (e.g., intersection of two sets, union of two sets, relative complement, absolute complement, etc.). Think of this as all the different ways you could color the segments of this figure (there are four segments, so 2^4^ ways of coloring) using only two colors, representing selection or rejection.

The immediate question is to ask why this might be relevant. The first part of the answer is that these colorings represent the full spectrum of binary logical operations (over two sets B and C that are both subsets of a population A). If selection is important, then to be able to deploy processes that manipulate selections hierarchically or in sequence seems critical. The brain would need the ability to not only process the content of a selected group of inputs (e.g., via convolutional methods) but also to manipulate the extent of that selection. There is no reason to think that all of these logical operations are actually implemented in the brain, nor that they should be in either the brain or in a machine. However, there is evidence to think that some are and, certainly, there is much to support that the operations of one-of-many, some-of-many, and none-of-many are indeed parts of actual attentional processes. In the examples that follow, these three different operations are illustrated. Importantly, the examples make clear that each experimental setup involves more than one operation.

**Figure 3:**
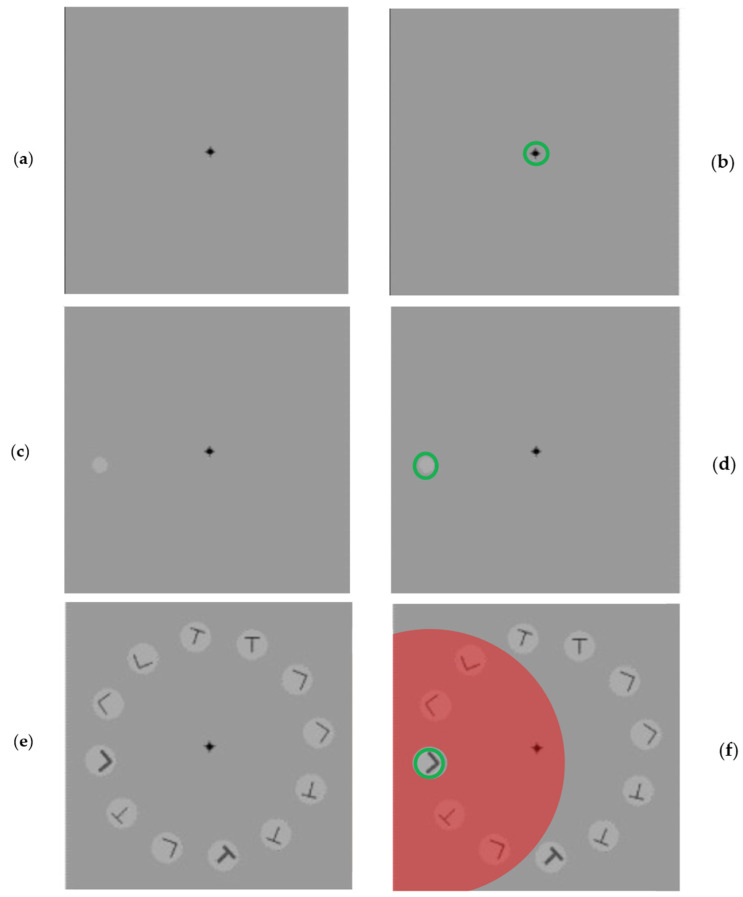
The attentional selections in the experiment of Cutzu and Tsotsos [25]. The left panel of figures show the sequence of displays presented to a subject. The right panel shows the attentional operation, with green indicating selection and red indicating suppression. (**a**) The first display gives the point to which subjects are instructed to fixate. (**b**) Shows that, in order to fixate, one must first attend that location (one-of-many). (**c**) The display gives the subject the cue to which to attend, while maintaining fixation. The cue is a region and thus a *some-of-many* selection is applied. (**d**) Shows the cue selection. (**e**) The test display with target and probe in bold. (**f**) Attending the cue location imposes a top-down suppressive surround shown in red; a none-of-many selection applied together with the some-of-many selection for the cue (while still fixating, of course) (adapted from [25]).

The first example is taken from the experiment in [25] that examined the prediction that spatial attention involves a suppressive surround. The context problem described in [26] appears whenever there is more than a single stimulus element within a single receptive field and this solution to it was proposed in [23]. Human subjects solve this using a top-down attentional suppressive surround and this has been well-documented [25,26,27,28,29]. Figure 3 shows an example from a published experiment [25]. It should be noted that these examples fall into Marr’s implementation level; they are human behaviors. They show how the complexity level qualification is orthogonal to Marr’s three levels and applies at each. The selection concepts themselves are still at the logic of the strategy level.

The left panel of figures show the sequence of displays presented to a subject. The right panel shows the attentional operation, with green indicating selection and red indicating suppression. There are three applications of different attentional process shown in Figure 3b,d,f. This example also shows that suppression is not restricted to distractors alone because that would mean you have already determined which stimulus is the target and which is the distractor, thus making accuracy extremely high (or perfect); it is not so observed. Since it is not observed this way, the suppression must be within the receptive field selected for top-down scrutiny (see [28]). It is important to highlight that, although the figure shows three observable attention examples, there are unobservable applications of attention here as well. Part of the subject’s instructions are to sequence their attention and to shift internal attention from the fixation point, guided by the onset of the cue, while maintaining the physical fixation behavior. For Figure 3e, there are many onsets yet again; fixation behavior is to be maintained and the onsets ignored as an eye movement cue. These are attentive selections of the none-of-many kind. Overall, during the time course of a single trial (about 300 ms), we see at least five instances of specific attentional behavior. In other words, this experimental trial involves a sequence of five different attentional acts so any logic of strategy, in Marr’s terms, that explains the observations must also include them.

**Figure 4:**
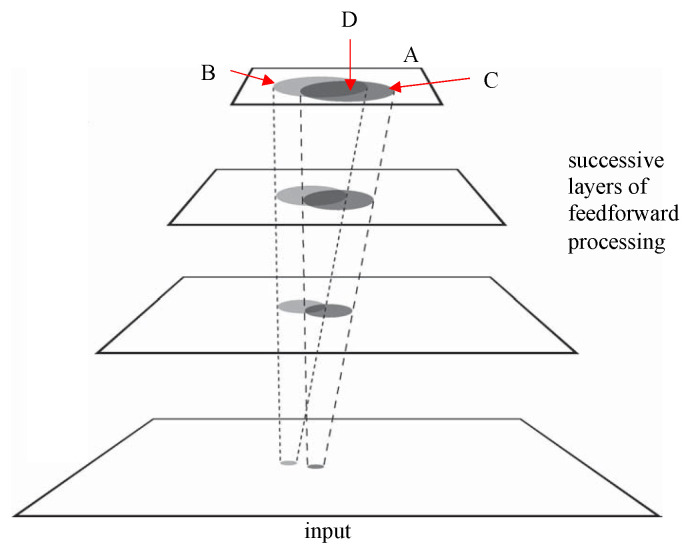
Crosstalk example. The figure illustrates the spatial region within in which the overlap makes an impact (region D in the top layer but this region evolves layer by layer from the input forward). The result is that, for two stimuli in the input, three regions with potentially different interpretations appear not only at the top layer (where they are labelled as B, C, and D and red arrows identify them), but in each layer. In other words, the input signal is rendered ambiguous. Attentional processes that suppress the interference in a top-down traversal, subsequent to the initial feedforward traversal, ameliorate this problem [23]. The spacing of stimuli clearly matters; the number of input stimuli also matters. (Adapted from [31], where the algorithm for how to realize such a top-down suppression is given).

A second example shows that selection operations span layers of a network. Earlier, it was mentioned how a sequence of selections can form a hierarchal representation, and how such hierarchies are important for ameliorating some of the complexity issues; in other words, this is not an implementation level issue and we remain at the computational level of analysis. The crosstalk problem detailed in [26] (often described later as the entanglement problem in deep neural networks, e.g., [30]), can be solved by suppressing the interfering contextual elements of a scene throughout the levels of this network. Figure 4 illustrates this. Crosstalk occurs whenever there are two or more separate visual events in the visual field. Each stimulus (two are shown in the input layer of the four-layer network of the figure) will activate a feed-forward cone of other neurons in the network, directly because the output of neurons in the visual cortex have one-to-many mappings from the lower layer to the higher layer (a diverging feedforward neural connectivity pattern) that overlap. The region of overlap will necessarily contain units whose activity is a function of both events (pooling response in the brain in attentive experiments has been shown to approximate the average not maximum of responses; [31]). Thus, each event interferes with the interpretation of other events in the visual field.

These examples show that there can be several different attentional mechanisms at play during any one experiment and these need to be teased apart in order for the full characterization to be possible. A nice side-effect of thinking of selection in this manner is that it provides a formalism for how to express set selection concepts. I note that the three classes of attentional mechanisms in my Selective Tuning model are selection (one-of-many), restriction (some-of-many), and suppression (none-of-many), each class having a number of specializations [10,31].

All of these possibilities for how selection might appear have a singular purpose: to improve the search through the space of possible interpretations and behaviors, both by reducing the size of a search space and reducing interference among elements within that search space. If you are wondering about the interference reduction aspect, consider that you use exactly this function every time you use your mobile phone. Adaptive beamforming is a well-understood methodology that performs signal processing by dynamically manipulating the combination of signals (how they interact and interfere with each other) so that the signal strength to and from a chosen direction is enhanced, while those to and from other directions are degraded. This is commonly used in cellular communication. Adaptive beamforming seeks to maximize the signal–to-interference-plus-noise ratio S/(I + N) and is proposed to be integral to the executive control of attentive behavior [32]. The real question that arises when thinking about selection in these terms is: For any given experiment that has been reported in the literature, regardless of human or animal subject, which of these operations might be at play and in which order? It is certainly not the case that attentional selection occurs only in the input space. Could it be that part of the confusion in the literature is due to assuming too coarse an interpretation of attentional action? 

The attention literature includes many expressions of frustration over the years, with two of my favorites being “Attention is in disarray” [33] and “After many thousands of experiments, we know only marginally more about attention than about the interior of a black hole” [34]. The literature also includes many expressions of certainty, such as “On attention itself, it is needless to discourse at length; its nature and conditions are familiar to every thoughtful student” [35], “Everyone knows what attention is” [36] and “Attention allows you abstract out a lot of irrelevant details and focus on what matters. Being able to pick out the relevant elements – that is what attention does.” [37]. Over-simplifications such as these on either side are not useful. 

The problem of attention necessarily involves a sequence of processing steps in both visual function and functional vision scenarios. Theories or models of attention should be able to account for such a sequence of attentional acts, how it may be realized, and how it is deployed.

## 4. The Current First Principles of Attention Are Too Simple

The main points of Section 2 and Section 3 seem to have broad agreement in the community. In important ways, they may be regarded as the First Principles of Attention, version 1.0. However, I feel they are now too simple, too abstract. It is likely that, as a community, we needed these simpler concepts decades ago to guide further exploration of these ideas. What may have been useful as a guide decades ago, now needs a deeper, less abstract, interpretation because it no longer serves the same purpose.

This is the reason to consider a deeper interpretation more in line with what Marr saw as his first level of analysis, the computational level. The point is to provide a description of the problem independent of algorithms, representations, and realizations: a description that applies to any implementation of a visual system. In Marr’s world, any implementation would be a solution to the computational level problem description. He assumed the goal of optimality when seeking representations or implementations. However, the inclusion of my complexity level analysis tells us that the tractability of the computational level problem is also important. The formal proofs mentioned earlier show that optimality in this domain is infeasible. Any given problem specification is inherently too difficult to solve completely and optimally and thus any realization can, at best, address only a portion of it. As such, complexity level analysis might be considered as a qualifier to Marr’s three levels: it informs the nature of solution that might be realizable in actual embodiments at any of the three levels.

A version 2.0 of the First Principles of Attention would include:

a. The necessity for attentional processing is present in any intelligent agent with non-trivial sensory abilities. Although it has been thought that the volume of an instance of sensory input is the direct cause, the grouping combinatorics of the elements of that instance are the real problem that attention must solve.
b. The problem of too much sensory input can be dealt with by utilizing knowledge or task direction in order to both reduce the amount but also to reduce the combinatorial relationships among input data.
c. The space of all possible input instances must be partitioned into subsets, each solvable perhaps with different methods with potentially differing levels of performance (accuracy as well as time to complete). The discovery of the most appropriate partitioning is a major challenge.
d. The commonly accepted act of attentional selection is too limited. It can be usefully expanded to include different kinds of selection in a set-theoretic, logical manner, such as selection of one-of-many, some-of-many, and none-of-many. These can be combined to create more complex selections and even sequenced so that hierarchies of representations are possible. Within a hierarchy of representations, the signal interference caused by converging neural pathways must be addressed.
e. Sequences of attentional actions are common and appear in almost any experiment and in all aspects of everyday life. It is quite unlikely that all the possible sequences are hardwired, suggesting that some form of executive is required to decide what elements are included, how they might be parameterized, how each is deployed, how the sequence is monitored for its impact, and how it might be repaired under failure.
f. Both the processing of a sensory instance and the corresponding response require attentional processing.
g. The multiple attentional behaviors require dynamic coordination and management tailored to the current interaction with the visual environment. Embodiment matters.

These each point to the need for a less abstract level of research in order to make progress. It is likely that most experimental investigations are actually describing real attention, but they are not all describing the same attentional act. It is true that perhaps all attentional acts are covered by an umbrella form of the term selection, but the previous discussion has shown that we can be more fine-grained about selection. The conclusions drawn and all the thoughtful attempts at explaining or accounting for the results observed might have limited value otherwise. If an observation is the result of two or more separate attentional events, it might be that an explanation that assumes a single event at the outset is not valid; it is an attentional conflation. If, on the other hand, the proper sequence of attentional events is asserted, any proposed conclusions will be more useful.

We also need integrative perspectives; we need to encourage (and reward) carefully justified big-picture frameworks that unify observations as arising from within the same computing substrate, whether that be biology or machine. The reason for the reward is that these are inherently riskier because they would involve a predictive component that, when tested, would reveal the framework’s validity. In other words, there must be incentives to permit failure of well-justified ideas.

How might these impact an attention research program? In many ways; some examples follow. A task-agnostic or task-independent theory or model is insufficient because it would not be scalable. Reliance on a single attentional effect, such as enhancement, is likely insufficient. Moving away from two-dimensional controlled settings, even if simulations are high quality, and focusing more on real world interactions allow the open problems of how to plan, synchronize, monitor, and evaluate the impact of attentional actions to become much more visible.

## 5. Discussion

There are several aspects of what is presented in this paper that deserve further discussion. The question of whether the kind of back-of-envelope computations as presented in Section 2 are relevant has been asked many times. If they stood alone, the question would be a very sensible one. However, they do not stand alone. These are illustrations that drive home the point of the theory that underlies them. Many authors have provided formal proofs of the computational difficulty of a large diversity of problems within perception, cognition, language, planning, and more. These highlight the fact that almost all non-trivial problems that cognitive science or artificial intelligence have considered are computationally very difficult (or intractable) in a formal sense. In practice, this means that it is unlikely that a single solution, optimal and correct for all possible instances of the problem, will ever be found. They point to the necessity of thinking about approximate solutions, heuristic approaches that are satisficing, dividing the space of possible instances into smaller chunks that may be more amenable to good solutions, tradeoffs between processing time and computer power, and similar tactics for managing computational complexity. None of this is new. Such theoretical proofs and solution approaches within computer science have been presented since the early 1970’s; van Rooij [17] provides an excellent summary of a wide range of problems directly relevant to cognitive science, [38] give a great guide to the connections between cognition and complexity, and [10] focuses on vision (and these are only a few of many other sources). Attention in the form of sequences of different selection strategies plays the role of simplifying problems by partitioning the space of possibilities into smaller, more tractable groupings. Such sequences then naturally lead to issues of executive control (see [32]).

Although Section 2 and Section 3 present the two first principles of attention, many might think there is a third. That third concept that you will have seen from your high vantage point of the attention literature is that of a *processing bottleneck*. It is difficult to know whether its proponents meant this physically or metaphorically, or perhaps both. Physically, it would mean that the brain actually has some sort of neural restriction to the flow of information. In other words, some information is selected and passed to some subsequent process and some is not. Think of a physical wine bottle, which has a neck. Fill it, turn it upside down, and set a timer to measure how long it takes to empty. Now cut the neck off and repeat. Is the time not less? Now, try this again with another bottle but this time cut off the bottom first. You will add water at the bottom (now the top) to maintain the rate of flow at the bottle output. How much water are you using? It looks like the same as is pouring out. Cut off the neck and repeat. Now, the amount you add is again the same as is pouring out, but larger. Try changing the rate of input. The point is that a physical bottleneck causes a backup if the amount coming in is greater than the amount going out. The amount coming in is what the retinas provide. If a bottleneck reduces this, where does the rest go if the input is at a constant rate? See the problem? Without providing (at least) a second pathway, there is a difficulty. This might have indeed been a possible evolved solution to the combinatorial problems described earlier. However, it would seem unlikely that a hard-wired restriction of the information flow to solve one sort of problem would not be accompanied by some other strategy to ensure that the blocked data did not contain anything of interest or perhaps of immediate danger. This now puts us at the early vs. late selection debate of the late 1950s and 1960s. Metaphorically, a bottleneck eventually allows all of the information to flow through and it just changes the rate of flow. This would not work for vision because changing the rate of information flow would only cause a backup, as new information presented to the sensory system comes at a fixed rate. The bottleneck concept might have been another one of those that had a utility for one period of time but is now outdated.

The issue of sampling a vast and somewhat unknown population of possible images and image sequences, as discussed earlier, is now being addressed by new efforts to harness sufficient computing resources to develop simulators that might generate appropriate numbers of samples (e.g., [39]). I have no doubt that impressively huge arrays of computer hardware will be deployed and unheard-of data set sizes will be created, but will they approach the scales presented earlier? This is uncertain, just as it is uncertain how these simulators will deal with the effects of imaging geometry. Will each scene need to be imaged by a camera placed at all possible vantage points and with all possible settings of each of its intrinsic parameters (e.g., focal length, shutter speed, gain, aperture, white balance) as well as all possible lighting configurations? If not, how will these imaging dimensions be sampled? It may be that neural network generalization might not compensate sufficiently (e.g., [40,41]). That is the visual function side; what about the functional vision side? The idea of sufficiently powerful and extensive simulation of the data seems to be an effort that is doomed for the general case, although it might suffice for very specific and narrow application domains. It does not take into account the very human ability to choose what stimulus to consider next based on current context. Further, the use of humanly infeasible amounts of training data cannot tell us much about the process of functional human vision.

## 6. Conclusions

Finally, after your long walk through the piles of papers on the plain, you arrive at the horizon. It was not an easy journey at all and you hesitate just a second before taking a peek beyond the horizon. Your mind is swarmed with all the new insights you have acquired. While moving your eyes for the next view you feel inspired, excited for the preview of what the next generation of thinkers might be developing. In the distance, there are many people at desks and in labs, all busy and sadly too far away for you to discern exactly what they are doing.

You awaken, open the curtains, and realize it is just another day. Normal routines dominate, searching your closet for what to wear, making breakfast after finding where your partner put the chocolate syrup you like on your pancakes, moving from room to room packing up what you need for your work day (where are those cookies I put aside for my snack?), taking care of family members’ needs (your child decides this is the perfect time for hide-and-seek in order to delay going to school!). All the while, you remember your mountaintop adventure and realize how many times you are choosing where to look (that is what hide-and-seek is all about), how often what you do is guided by familiarity with your home layout and contents, how often you need to look at exactly what you need to grasp before doing so in order to get a good hold and not drop it, and so much more. You recall from those many papers you read how you learned about experiments dealing with all of these and other aspects of your everyday activities. As you step into your car, you wonder exactly how do you make sure the road is always clear ahead, how do you plan your next turn, how do you…? You have endless questions. You marvel at how seamlessly these abilities and activities come together as evidenced by your own smooth and effortless behavior. The gist of the image from the horizon, now fading from memory, suddenly becomes clear: attention is the result of an intelligent collection of selection processes that, when dynamically coordinated in a purposeful manner, tame any difficulties that may be caused by the combinatorics of the scenes and tasks you face every day. This is just as valid for a biological brain as it is for a machine brain. It is the mosaic picture to which everyone beyond the horizon was adding tiles.

**Epilogue**

In the end, perhaps this walk down from the mountaintop was a tough one. It would be very reasonable to wonder exactly where the many well-known models and theories fit. Moreover, what about the many mountains of experimental data on visual attention? There are several sources where one can look for these and many others were mentioned earlier ([18,42,43,44,45,46,47,48], and on the computational side [3,31,32,49,50,51,52,53,54,55,56], as well as the excellent coverage by others, such as [4,29,57,58] and more).

The importance of this massive past effort is not questioned but it does not contribute to the goals of this paper. In this opinion piece, I tried to address the generic nature of the problem of attention to show that a formal, computational problem actually exists, without relying (as little as possible) on data or algorithms or representations for justification (although examples were shown as illustration). All papers on the topic of visual attention present data, propose explanations for how that data come about, discuss where and how the brain produces these data, lay out possible mathematics that capture this process, and so on. They try to cover all of Marr’s levels at once. The flaw here is that, at best, they describe only one small portion of the data, usually do not characterize whether or how that portion may be solved, and then try to generalize to all the data. Recall the earlier discussions on tractability. It is almost as if they tacitly assume that the problem is how to explain a particular body of data. That is indeed a problem but not the problem I address here. Even if the body of data is large the intractability I described above means that it is hopeless to think one can ever assemble a sensible and fully representative population of data. This means one needs to think abstractly. On this point, Marr was right.

## Data Availability

Not applicable.
